# "Sinopharm", "Oxford-AstraZeneca", and "Pfizer-BioNTech" COVID-19 vaccinations: testing efficacy using lung CT-volumetry with comparative analysis of variance (ANOVA)

**DOI:** 10.1186/s43055-023-00999-x

**Published:** 2023-03-06

**Authors:** Ahmed Samir, Dina Altarawy, Rania Ahmed Sweed, Amr A. Abdel-Kerim

**Affiliations:** 1 Department of Radio-Diagnosis, Faculty of Medicine, Alexandria University, Alexandria, Egypt; 2 Department of Chest Diseases, Faculty of Medicine, Alexandria University, Alexandria, Egypt

**Keywords:** COVID-19, Vaccination, Sinopharm, Oxford-Astrazeneca, Pfizer-BioNTech, CT-Volumetry

## Abstract

**Background:**

Several clinical studies tested the efficacy of the different COVID-19 vaccinations while very few radiological researches targeted this issue before.

**Aim of the work:**

To verify the additive role of lung CT-Volumetry in testing the efficacy of three widely distributed COVID-19 vaccinations; namely the "Sinopharm", "Oxford-AstraZeneca", and "Pfizer-BioNTech" vaccinations, with comparative analysis of variance (ANOVA).

**Results:**

This study was retrospectively conducted on 341 COVID-19 patients during the period between June/2021 and March/2022. Based on the immunization status, they were divided into four groups; group (A) included 156/341 (46%) patients who did not receive any vaccination (control group), group (B) included 92/341 (27%) patients who received "Sinopharm" vaccine, group (C) included 55/341 (16%) patients who received "Oxford-AstraZeneca" vaccine, group (D) included 38/341 (11%) patients who received "Pfizer-BioNTech" vaccine. Every group was subdivided based on the medical history into three groups; group (1) patients without comorbidities, group (2) patients with comorbidities, and group (3) immunocompromised patients. Automated CT volumetry was calculated for the pathological lung parenchyma. Five CT-severity scores were provided (score 0 = 0%, score 1 = 1–25%, score 2 = 25–50%, score 3 = 51–75%, and score 4 = 76–100%). Analysis of variance (ANOVA) including Tukey HSD testing was utilized in comparison to the non-immunized patients. The "Phizer-Biontech" vaccine succeeded to eliminate severity in patients without and with comorbidity, and also decreased severity in immunocompromised patients (from 79 to 17%). The "Oxford-AstraZeneca" vaccine and to a lesser extent "Sinopharm" vaccine also decreased the clinical severity in patients with comorbidities and immunocompromised patients (from 15 to 9% & 10% as well as from 79 to 20% & 50% respectively). Significant variance was proved regarding the use of "Sinopharm", "Oxford-AstraZeneca", and "Phizer-Biontech" vaccines in patients without comorbidities (*f*-ratio averaged 4.0282, 10.8049, and 8.4404 respectively, also *p*-value averaged 0.04632, 0.001268, and 0.004294). Significant variance was proved regarding the use of "Oxford-AstraZeneca", and "Phizer-Biontech" vaccines in patients with comorbidities and immunocompromised patients (*f*-ratio averaged 4.7521, and 4.1682 as well as 11.7811, and 15.6 respectively, also *p*-value averaged 0.03492, and 0.04857, as well as both 0.003177, and 0.0009394 respectively, all < 0.05). No significant variance was proved regarding the use of the "Sinopharm" vaccine.

**Conclusions:**

In addition to the decline of clinical severity rates & CT severity scores, a significant variance was proved regarding the use of the "Sinopharm", "Oxford-AstraZeneca", and "Phizer-Biontech" vaccines in patients without comorbidities. Significant variance was also proved regarding the use of the "Oxford-AstraZeneca" and "Phizer-Biontech" vaccines in patients with comorbidities and immunocompromised patients. Despite that, no significant variance could be proved regarding the use of the "Sinopharm" vaccine in these patients, it decreases the percentage of clinical severity and CT severity scores.

## Background

Humanity has been suffering for three years during the COVID-19 pandemic. Millions of deaths were reported. The life of other human beings was threatened. The social and economic compromise was cruel. The health facilities were exhausted. Since then, various countries have worked hard to produce efficient vaccines [1]. They aimed to improve the survival of the infected patients as it is well known that the available COVID-19 vaccinations do not prevent infections or re-infections but decrease morbidity and mortality. They can alleviate the severity of symptoms, and reduce the need for hospital admission and the burden on community health facilities. Furthermore, they can limit the spread of infection from asymptomatic individuals [2–4].

Different types of vaccines with different mechanisms of action have been developed and approved for emergency use [5, 6]. They implemented mRNA vaccines such as Pfizer-BioNTech, and routine inactivated vaccines such as Sinopharm [7]. There was also "Oxford-AstraZeneca" ChAdOx1 nCoV-19 vaccine (AZD1222) which consists of a replication-deficient chimpanzee adenoviral vector ChAdOx1 that curries the SARS-CoV-2 structural surface glycoprotein antigen (spike protein; nCoV-19) gene [8].

The antibody titer and the impact on the clinical course were the main parameters that were utilized to determine the vaccination efficacy in several previous clinical studies [7].

The majority of the previous radiological studies focus on post-vaccination imaging pitfalls, especially in positron emission tomography (PET/CT) examinations. Meanwhile, very few previous radiological researches targeted the assessment of vaccination efficacy [9].

*Aim of the work:* To clarify the additive role of lung CT-Volumetry in testing the efficacy of three widely distributed COVID-19 vaccinations; namely "Sinopharm/BBIBP-CorVi", "Oxford-AstraZeneca/ChAdOx1 nCoV-19 (AZD1222)", and "Pfizer-BioNTech" vaccinations, with comparative analysis of variance (ANOVA).

## Methods

**Fig. 1:**
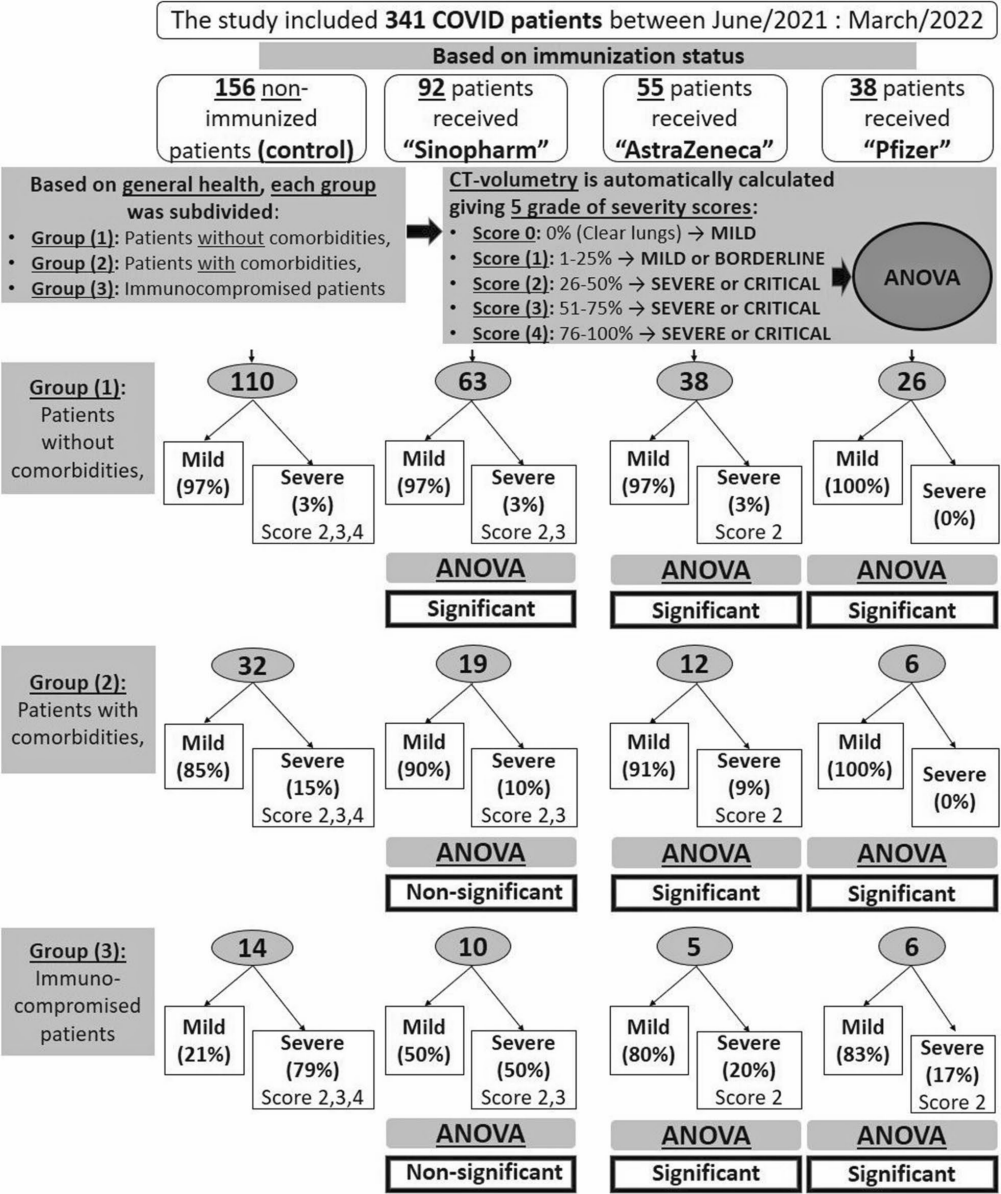
A flow-diagram is demonstrating the study design and methodology

A flow-diagram is demonstrating the study design, methodology, and brief results. (Fig. 1)

This study was retrospectively conducted on 341 patients, proved with COVID-19 infection by PCR testing, and were eligible for chest CT examinations, during the period between June/2021 and March/2022. They were divided based on the immunization status into four groups, including patients with negative history of vaccination (control group A), and patients who received a full regimen of either "Sinopharm" or "Oxford-AstrZeneca" or "Pfizer-BioNTech" vaccine (group B, C, and D). COVID-19 infection was proved after one month to six months from receiving a full vaccination regimen. Every group was furtherly subdivided based on patients' general health and medical history into three groups; group (1) including healthy subjects without comorbidities, group (2) including patients with chronic comorbidities, and group (3) including immuno-compromised patients. HRCT was performed for each group. Automated CT volumetry was calculated for the pathological lung areas. Five scoring grades were divided based on CT-volumetry score (score 0 = 0%, score 1 = 1–25%, score 2 = 25–50%, score 3 = 51–75%, and score 4 = 76–100%). Analysis of variance (ANOVA) including Tukey HSD testing was utilized.

*The inclusion criteria were as follows* (1) patients proved with COVID-19 infection by PCR testing and did not receive any kind of vaccination, and (2) patients proved with COVID-19 infection by PCR and received a full regimen of either "Sinopharm", or "Oxford-AstraZeneca", or "Phizer" vaccination one to six months before infection.

*The exclusion criteria were as follows* (1) poor quality of CT images because of patient tachypnea and unavoidable respiratory motion artifacts, (2) patients who did not complete the immunization regimen, and (3) patients infected during the first month after completing their vaccination regimen because of insufficient duration for antibodies development.

The study was approved by the "Institutional Ethics Committee". After assuring full respect for the confidentiality of the personal data and the medical records, the "Research Ethics Board" waived patient consent.

This manuscript does not overlap with any previously published works.

Three expert consulting radiologists and a single consulting pulmonologist participated in this study; the radiological experience ranged from 11 to 26 years while the clinical experience averaged 17 years. The radiologists worked together in a consensus and they were blinded from the clinical data and the immunization history.

### CT-scanning and CT-volumetry

The chest high-resolution CT (HRCT) examinations were performed on several multi-detector CT machines as follows: (1) SOMATOM Sensation 64, Siemens Medical Systems, Germany, (2) Canon Medical Systems; Toshiba Aquilion 64, Japan, and (3) Canon Medical Systems; Toshiba Aquilion CXL/CX 128, Japan.

The scanning parameters for the chest CT examinations were as follows: [1] The slice thickness: 1–1.25 mm, [2] The tube rotation: 0.6–0.9 s, [3] The detector Collimation 1 mm, [4] 120–130 kVp, and 200 mA, and [5] FOV = 350 mm × 350 mm. The examinations were performed without intravenous contrast administration.

The CT-Volumetry was carried out using OsiriX MD 11.0 software (Pixmeo SARL, Geneva, Switzerland), therefore the variability of the CT machines did not impact this quantitative assessment. An automated calculation of the pathological and total lung volumes was performed using the threshold interval adjustment during the region of interest (ROI) 2D/3D color-coded reconstruction. The interval for the total lung volume calculation ranged between (0:− 1024 Hu), meanwhile, the interval for the pathological lung volume estimation ranged between (0:− 700 Hu).

Five grades of severity were divided based on CT-volumetry score (score 0 = 0%, score 1 = 1–25%, score 2 = 25–50%, score 3 = 51–75%, and score 4 = 76–100%).

Morphologic CT-assessment was also performed based on the universal CT-signs of COVID-19 diagnosis, particularly the CT signs of severity including the “crazy-paving pattern” [9].

### Clinical contribution

The main clinical role was gathering a detailed history of the immunization status. Furtherly, a correlation with clinical course and outcome was carried out. It followed the universal criteria for the classification of clinical severity including; Oxygen saturation in room air (O2-RA), presence and grade of dyspnea, and respiratory rate (RR) [10]. Severe or critically ill patients with O2-RA < 93% and RR > 30/min were admitted to a hospital or even intensive care unit (ICU) receiving specific protocol of management including steroids, anti-viral drugs, and respiratory support starting from the initial high flow nasal oxygen therapy up to the mechanical ventilation [10].

### Statistical analysis

It included: (1) Estimation of the prevalence and ratios of the demographic factors, clinical severity, and CT severity scores. (2) Estimation of the mean, standard deviation (SD), normality, skewness, and excess kurtosis for each main and subgroup of patients in addition to the *f-*ratio*, p-*value, effect size, and η2 (variance from average) from the ANOVA analysis with Tukey HSD using an online calculator (https://www.https://www.statskingdom.com/180Anova1way.html). The *p*-value (< 0.05) was considered statistically significant.

## Results

Based on the immunization status, 341 COVID-19 patients were divided into four groups; group (A) included 156/341 (46%) patients who did not receive any vaccination before and were considered as the control group, group (B) included 92/341 (27%) patients who received two doses of "Sinopharm" vaccine, group (C) included 55/341 (16%) patients who received two doses of "Oxford-AstraZeneca" vaccine, group (D) included 38/341 (11%) patients who received a single dose of "Pfizer-BioNTech" vaccine. The patients in each group were furtherly subdivided based on the presence or absence of comorbidity or immunocompromised status.

The radiological and clinical severity generally accounted for 7 and 12.1% in the vaccinated and non-vaccinated patients respectively. Strikingly it was increased in immunocompromised patients compared to other patients without or with commodities (reaching 79% in non-vaccinated patients and ranges 17–50% in vaccinated patients).

### Patients with good general health and absent comorbidity

The "Phizer-Biontech" vaccine succeeded to eliminate severity in infected patients with good general health and absent comorbidity.

Despite that the clinical severity averaged 3% in the non-immunized patients as well as the "Sinopharm" received patients and the "Oxford-AstraZeneca" received patients, the CT severity scores had declined in the "Oxford-AstraZeneca" received patients (score 2) and the "Sinopharm" received patients (score 2–3) in comparison to the non-immunized patients (reached score 4).

This is detailed as follows:

#### Non-immunized patients (control group)

They were 110/156 (71%) patients. Their age ranged from 18 to 56 years with a male-to-female ratio of 3:2. (Table 1) Clinically, 107/110 (97%) patients were mild. By CT-volumetry, 25 (23%) patients scored (0) and 82 (74%) patients scored (1). Meanwhile, 3/110 (3%) patients were clinically severe or critical, showed a "crazy paving pattern" in HRCT, and were admitted to the hospital. one (1%) patient scored (2), one (1%) patient scored (3) and one (1%) patient scored (4). (Tables 1, 2).

**Table 1:** Demographic results, types of comorbidities and severity classifications in each group of patients

Total	Non Immunized	Sinopharm	Oxford-AstraZeneca	Pfizer-BioNTech
	156	92	55	38
*Patients with good general health and absent co-morbidity*				
Age	18–56y	29–69y	30–69y	35–67y
Sex	M:F = 3:2	M:F = 2:1	M:F = 4:1	M:F = 1:1
Comorbidity	–	–	–	–
Mild clinical course	107 (97%)	61 (97%)	37 (97%)	26 (100%)
Hospitalization for severe or critical patients	*3 (3%)*	*2 (3%)*	*1 (3%)*	*0*
“Crazy-paving” CT-sign of severity	3	2	1	0
Total	110 (100%)	63 (100%)	38 (100%)	26 (100%)
*Patients with chronic co-morbidity*				
Age	39–71y	41–73y	41–70y	43–69y
Sex	M:F = 1:3	M:F = 2:3	M:F = 1:3	M:F = 1:2
Comorbidity:	* COPD: 3 * DM: 15 * Cardiac: 11 * Rheumatologic: 2 * Liver cirrhosis: 4 * Renal failure: 2	* COPD: 2 * DM: 9 * Cardiac: 5 * Rheumatologic: 7 * Liver cirrhosis: 2 * Renal failure: 2	* COPD: 3 * DM: 5 * Cardiac: 6 * Rheumatologic: 0 * Liver cirrhosis: 0 * Renal failure: 0	* COPD: 1 * DM: 4 * Cardiac: 4 * Rheumatologic: 0 * Liver cirrhosis: 0 * Renal failure: 0
Mild clinical course	27 (85%)	17 (90%)	11 (91%)	6 (100%)
Hospitalization for severe or critical patients	*5 (15%)*	*2 (10%)*	*1 (9%)*	*0*
“Crazy-paving” CT-sign of severity	5	2	1	0
Total	32 (100%)	19 (100%)	12 (100%)	6 (100%)
*Immunocompromised patients*				
Age	21–67y	34–79y	41–76y	39–73y
Sex	M:F = 2:3	M:F = 1:4	M:F = 1:4	M:F = 2:3
Malignancy ± CRT	14	10	5	4
Mild clinical course	3 (21%)	5 (50%)	4 (80%)	5 (83%)
Hospitalization for severe or critical patients	*11 (79%)*	*5 (50%)*	*1 (20%)*	*1 (17%)*
“Crazy-paving” CT-sign of severity	11	5	2	2
Total	14 (100%)	10 (100%)	5 (100%)	6 (100%)

**Table 2:** CT-Severity scores in each group of patients according status of immunization and general health with ANOVA

Total	Non Immunized	Sinopharm	Oxford-AstraZeneca	Pfizer-BioNTech
	156 (46%)	92 (27%)	55 (16%)	38 (11%)
*Patients with good general health and absent co-morbidity*				
Score 0 (Clear lungs)	25 (23%)	35 (56%)	27 (71%)	18 (69%)
Score 1 (1–25%)	82 (74%)	26 (41%)	10 (26%)	8 (31%)
Score 2 (26–50%)	1 (1%)	1 (1.5%)	1 (3%)	0
Score 3 (51–75%)	1 (1%)	1 (1.5%)	0	0
Score 4 (76–100%)	1 (1%)	0	0	0
Mean	0.69091	0.49206	0.31579	0.30769
Standard deviation	0.63166	0.61887	0.52532	0.47068
Normality	1.066e−14	2.368e−10	7.953e−9	1.798e−7
Skewness	1.467299	1.295957	1.403173	0.885246
Excess kurtosis	6.718522	2.725637	1.126072	− 1.324728
Total	110 (100%)	63 (100%)	38 (100%)	26 (100%)
*Patients with chronic co-morbidity*				
Score 0 (Clear lungs)	5 (16%)	2 (11%)	7 (58%)	4 (67%)
Score 1 (1–25%)	22 (69%)	15 (79%)	4 (33%)	2 (33%)
Score 2 (26–50%)	2 (6%)	1 (5%)	1 (9%)	0
Score 3 (51–75%)	1 (3%)	1 (5%)	0	0
Score 4 (76–100%)	2 (6%)	0	0	0
Mean	1.15625	1.05263	0.5	0.33333
Standard deviation	0.95409	0.62126	0.6742	0.5164
Normality	4.273e–7	0.000008665	0.001741	0.005544
Skewness	1.80862	1.527857	1.067933	0.968246
Excess kurtosis	3.88361	5.618255	0.352	− 1.875
Total	32 (100%)	19 (100%)	12 (100%)	6 (100%)
*Immunocompromised patients*				
Score 0 (Clear lungs)	0	1 (10%)	3 (60%)	4 (67%)
Score 1 (1–25%)	3 (21%)	4 (40%)	1 (20%)	1 (17%)
Score 2 (26–50%)	5 (36%)	2 (20%)	1 (20%)	1 (17%)
Score 3 (51–75%)	4 (29%)	2 (20%)	0	0
Score 4 (76–100%)	2 (15%)	1 (10%)	0	0
Mean	2.35714	1.8	0.6	0.5
Standard deviation	1.00821	1.22927	0.89443	0.83666
Normality	0.09644	0.4046	0.06792	0.01418
Skewness	0.19301	0.46656	1.257788	1.536722
Excess kurtosis	− 0.819417	− 0.543592	0.3125	1.428571
Total	14 (100%)	10 (100%)	5 (100%)	6 (100%)

#### "Sinophram" received patients

**Fig. 2:**
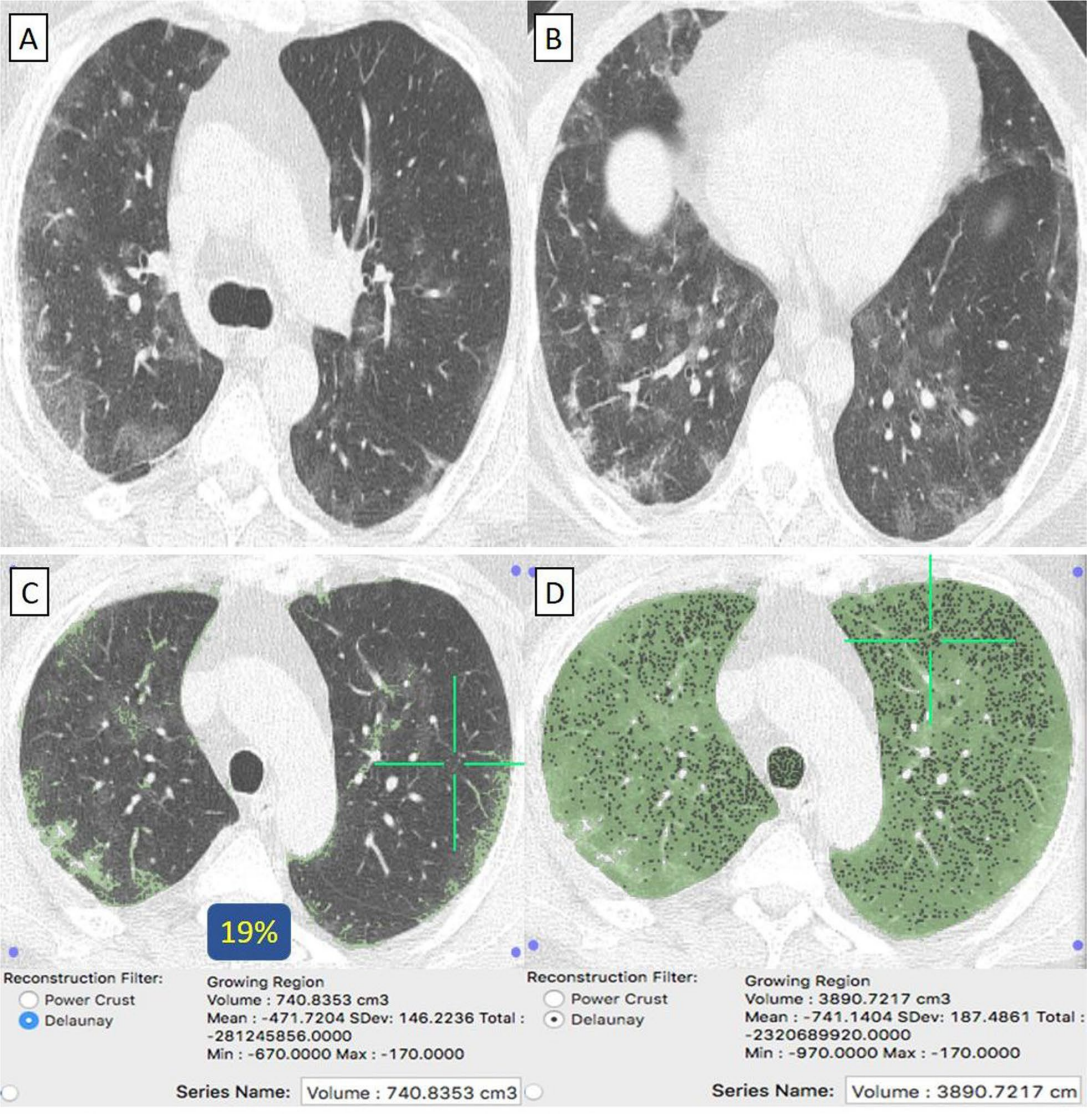
A 58-year-old female patient with negative past medical history and had received “Sinopharm” vaccine then got COVID-19 infection. **A**, **B** Axial chest CT cuts (lung window) showed bilateral mainly peripheral located ground-glass pneumonic patches and minimal right pleural reaction. **C** CT-Volumetry of the pathological lung parts averages 740 cc. **D** CT-Volumetry of total lung volume averages 3890 cc. So, only 19% of the lung parenchyma was diseased (Score 1)

**Fig. 3:**
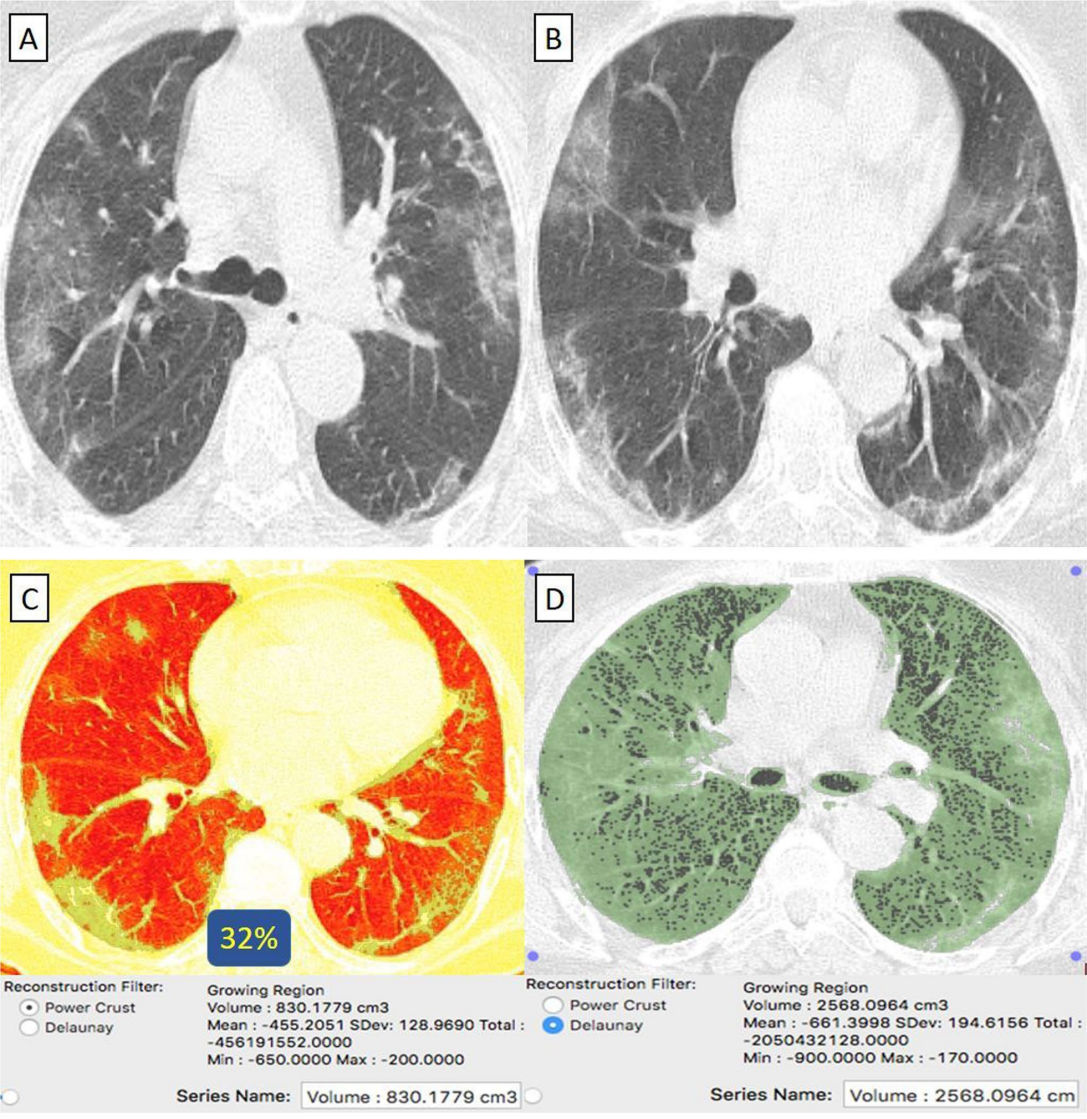
A 55-year-old female patient with negative past medical history and had received “Sinopharm” vaccine then got COVID-19 infection. **A**, **B** Axial chest CT cuts (lung window) showed bilateral mainly peripheral located ground-glass pneumonic patches with peripheral sub-pleural sparing (Atol-sign). **C** CT-Volumetry of the pathological lung parts averages 830 cc. **D** CT-Volumetry of total lung volume averages 2568 cc. So, 32% of the lung parenchyma was diseased (Score 2)

They were 63/92 (68%) patients. Their age ranged from 29 to 69 years with a male-to-female ratio of 2:1. (Table 1) Clinically, 61/63 (97%) patients were mild. By CT-volumetry, 35 (56%) patients scored (0) and 26 (41%) patients scored (1) (Fig. 2). Meanwhile, 2/63 (3%) patients were clinically severe or critical, showed a "crazy paving pattern" in HRCT, and were admitted to the hospital. One (1.5%) patient scored (2) (Fig. 3), and one (1.5%) patient scored (3). (Tables 1, 2).

#### "Oxford-AstraZeneca" received patients

**Fig. 4:**
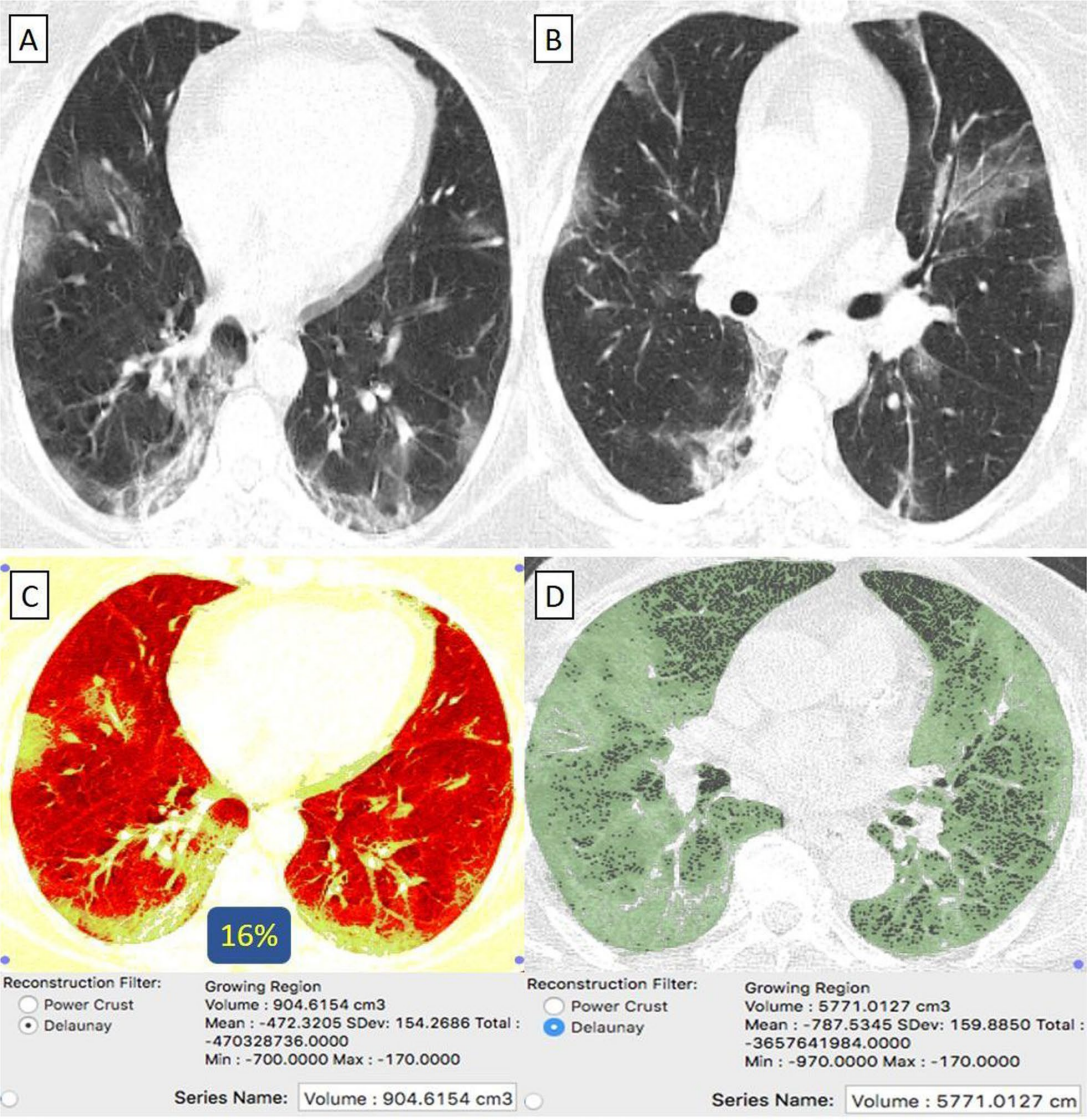
A 43-year-old female patient with negative past medical history and had received “Oxford-AstraZeneca” vaccine then got COVID-19 infection. **A**, **B** Axial chest CT cuts (lung window) showed bilateral mainly peripheral located ground-glass pneumonic patches and fine atelectatic bands. **C** CT-Volumetry of the pathological lung parts averages 904 cc. **D** CT-Volumetry of total lung volume averages 5771 cc. So, only 16% of the lung parenchyma was diseased (Score 1)

They were 38/55 (69%) patients. Their age ranged from 30 to 69 years with a male-to-female ratio of 4:1. (Table 1) Clinically, 37/38 (97%) patients were mild. By CT-volumetry, 27 (71%) patients scored (0) and 10 (26%) patients scored (1) (Fig. 4). Meanwhile, 1/38 (3%) patients was clinically severe, showed a "crazy paving pattern" in HRCT, were admitted to the hospital, and scored (2). (Tables 1, 2).

#### "Pfizer-BioNTech" received patients

**Fig. 5:**
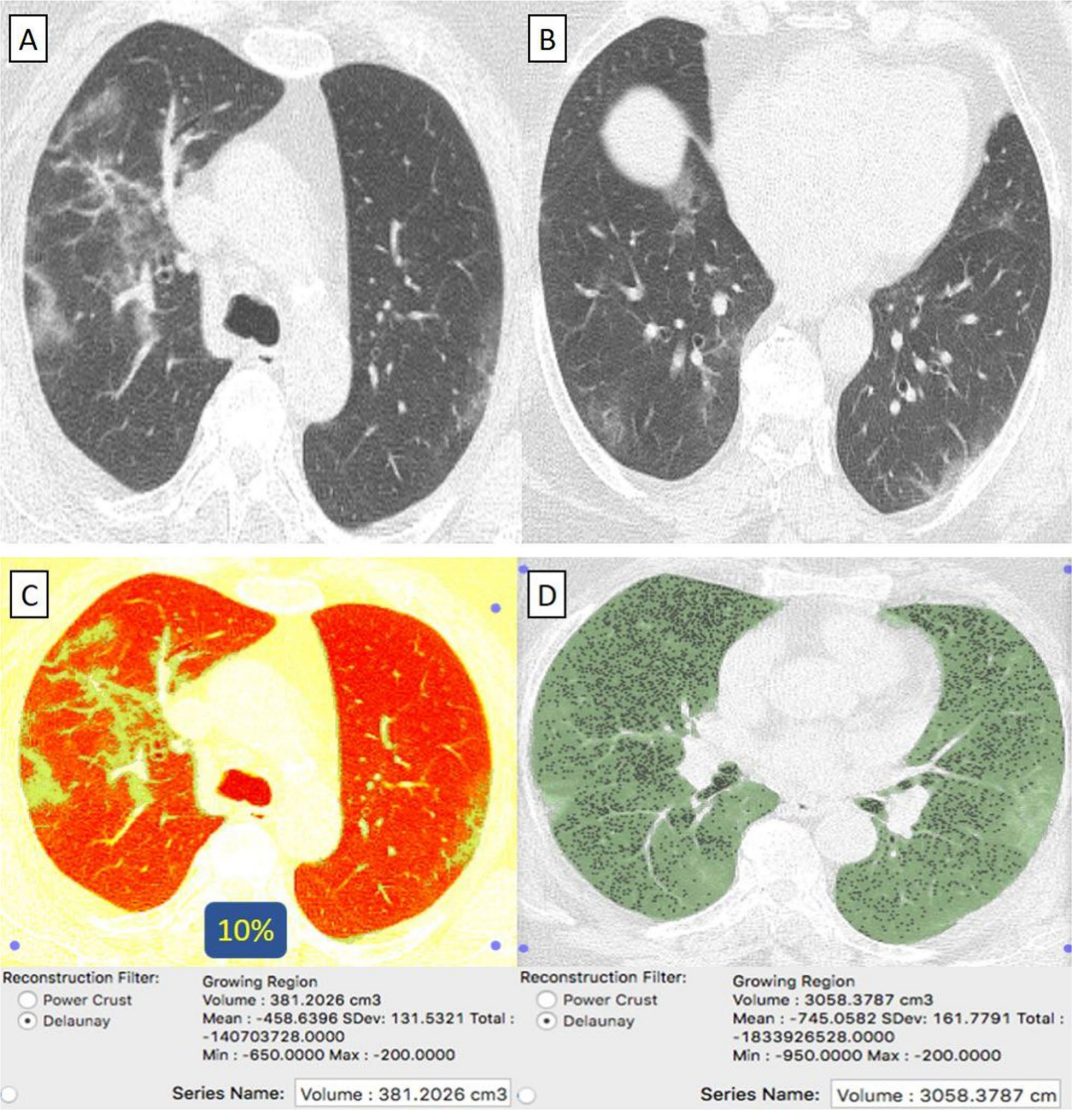
A 58-year-old female patient with negative past medical history and had received “Phizer-Biontech” vaccine then got COVID-19 infection. **A**, **B** Axial chest CT cuts (lung window) showed bilateral mainly peripheral located ground-glass pneumonic patches. **C** CT-Volumetry of the pathological lung parts averages 381 cc. **D** CT-Volumetry of total lung volume averages 3058 cc. So, only 10% of the lung parenchyma was diseased (Score 1)

They were 26/38 (68%) patients. Their age ranged from 35 to 67 years with a male-to-female ratio of 1:1. (Table 1) All patients were clinically mild. By CT-volumetry, 18 (69%) patients scored (0) and eight (31%) patients scored (1) (Fig. 5). (Tables 1, 2).

### Patients with comorbidity

The "Phizer-Biontech" vaccine again succeeded to eliminate severity in infected patients with comorbidity.

The "Oxford-AstraZeneca" vaccine and to a lesser extent the "Sinopharm" vaccine also decreased the clinical severity (averaged 9% and 10% respectively), compared to 15% clinical severity detected in non-immunized patients. The CT severity scores also declined in the "Oxford-AstraZeneca" received patients (score 2) and the "Sinopharm" received patients (score 2–3) in comparison to the non-immunized patients (reached score 4).

This is detailed as follows:

#### Non-immunized patients (control group)

They were 32/156 (21%) patients. Their age ranged from 39 to 71 years with a male-to-female ratio of 1:3. The classification of comorbidities was described in (Table 1). Clinically, 27/32 (84%) patients were mild. By CT-volumetry, 5 (16%) patients scored (0) and 22 (69%) patients scored (1). Meanwhile, 5/32 (16%) patients were clinically severe or critical, showed a "crazy paving pattern" in HRCT, and were admitted to the hospital. Two (6%) patients scored (2), one (3%) patient scored (3) and two (6%) patients scored (4). (Tables 1, 2).

#### "Sinophram" received patients

**Fig. 6:**
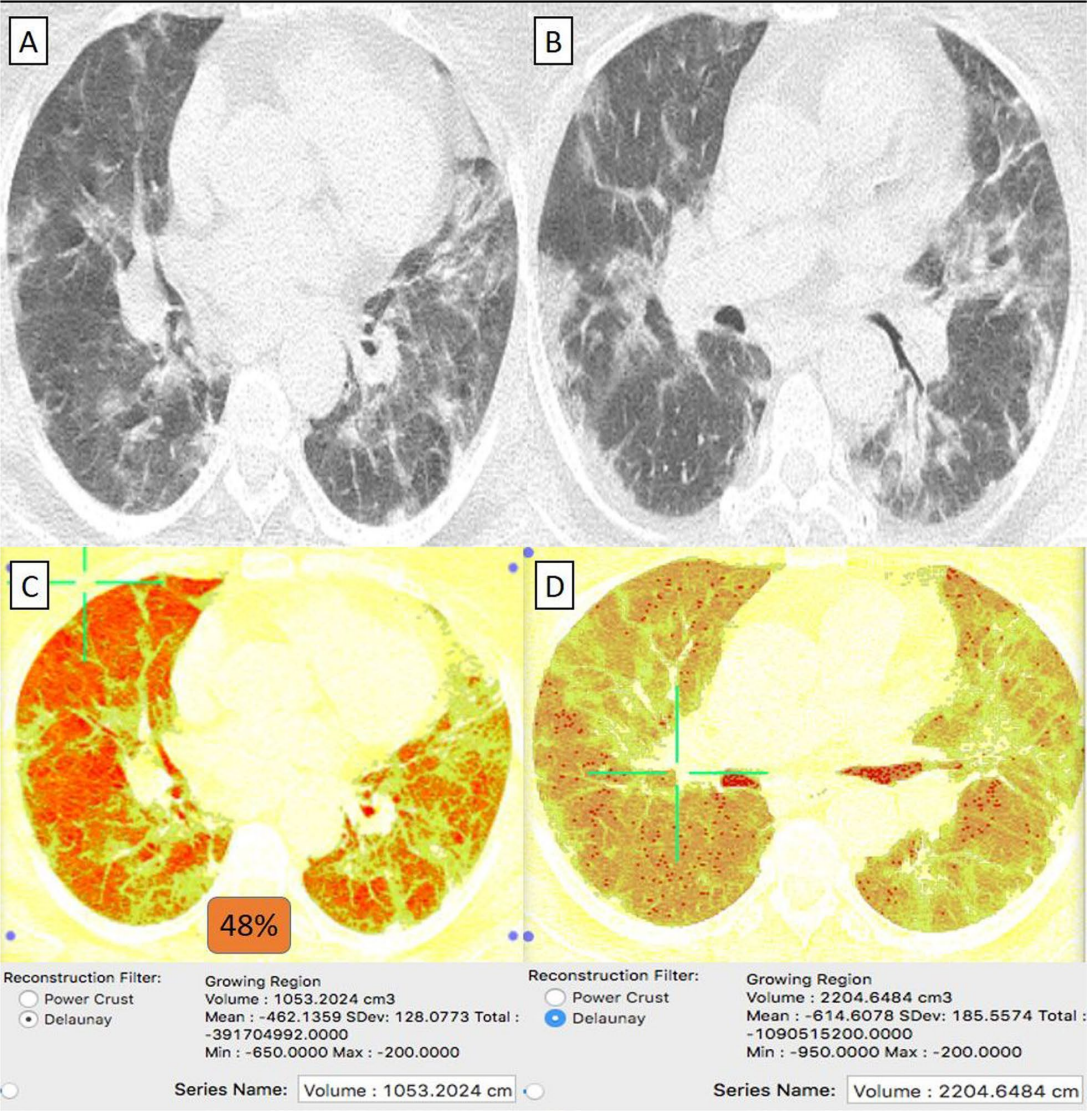
A 69-year-old female cardiac and diabetic patient who had received “Sinopharm” vaccine then got COVID-19 infection. **A**, **B** Axial chest CT cuts (lung window) showed bilateral mainly peripheral located and to lesser extent bronchocentric ground-glass pneumonic patches. **C** CT-Volumetry of the pathological lung parts averages 1053 cc. **D** CT-Volumetry of total lung volume averages 2204 cc. So, 48% of the lung parenchyma was diseased (Score 2)

**Fig. 7:**
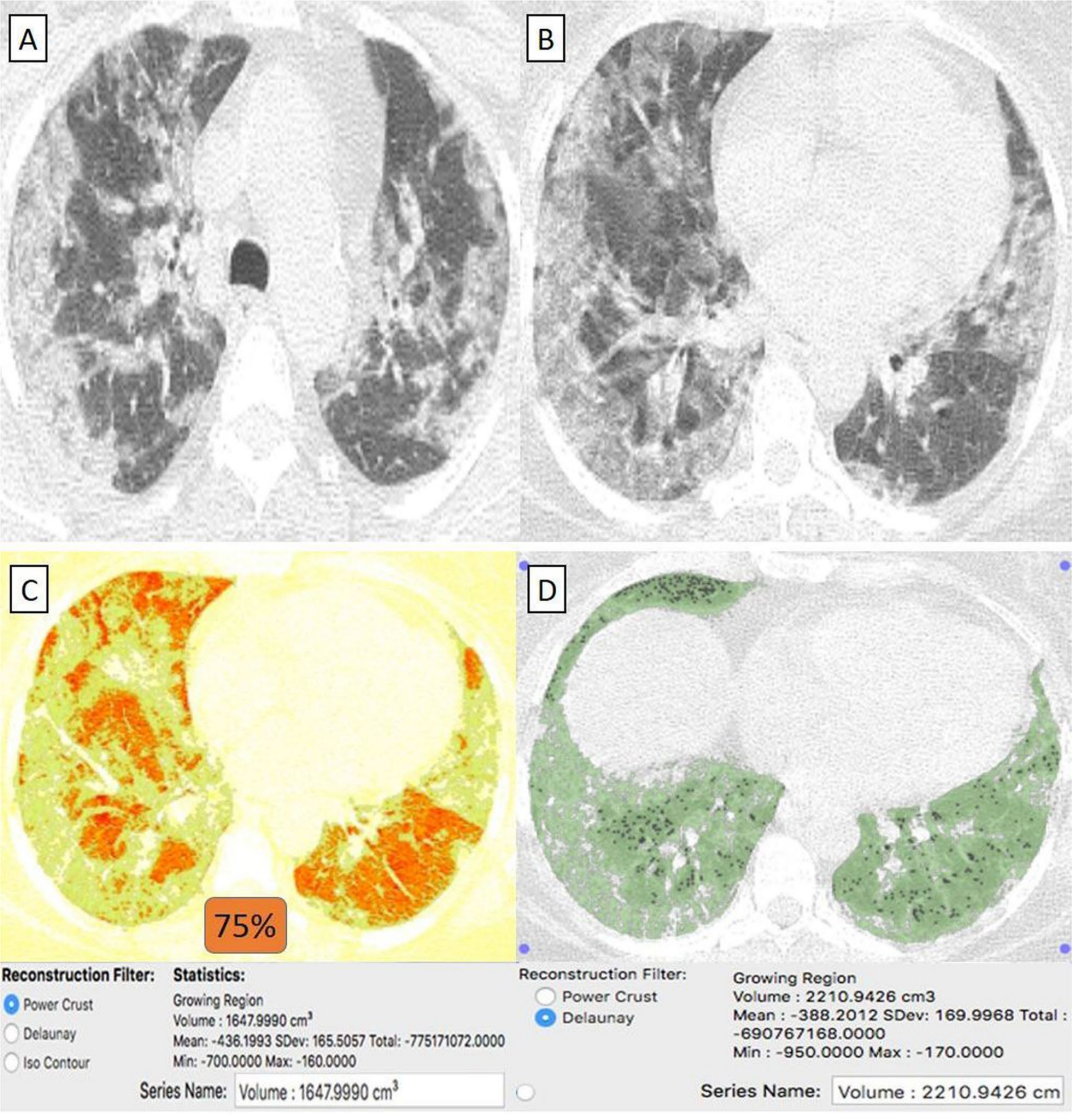
A 55-year-old female patient with history of systemic lupus erythematosus (SLE) and had received “Sinopharm” vaccine then got COVID-19 infection. **A**, **B** Axial chest CT cuts (lung window) showed bilateral mainly peripheral located wide spread ground-glass pneumonic patches. **C** CT-Volumetry of the pathological lung parts averages 1647 cc. **D** CT-Volumetry of total lung volume averages 2210 cc. So, 75% of the lung parenchyma was diseased (Score 3)

They were 19/92 (21%) patients. Their age ranged from 41 to 73 years with a male-to-female ratio of 2:3. The classification of comorbidities was described in (Table 1). Clinically, 17/19 (89%) patients were mild. By CT-volumetry, 2 (11%) patients scored (0) and 15 (79%) patients scored (1). Meanwhile, 2/19 (11%) patients were clinically severe or critical, showed a "crazy paving pattern" in HRCT, and were admitted to the hospital. One (5%) patient scored (2) (Fig. 6), and one (5%) patient scored (3) (Fig. 7). (Tables 1, 2).

#### "Oxford-AstraZeneca" received patients

**Fig. 8:**
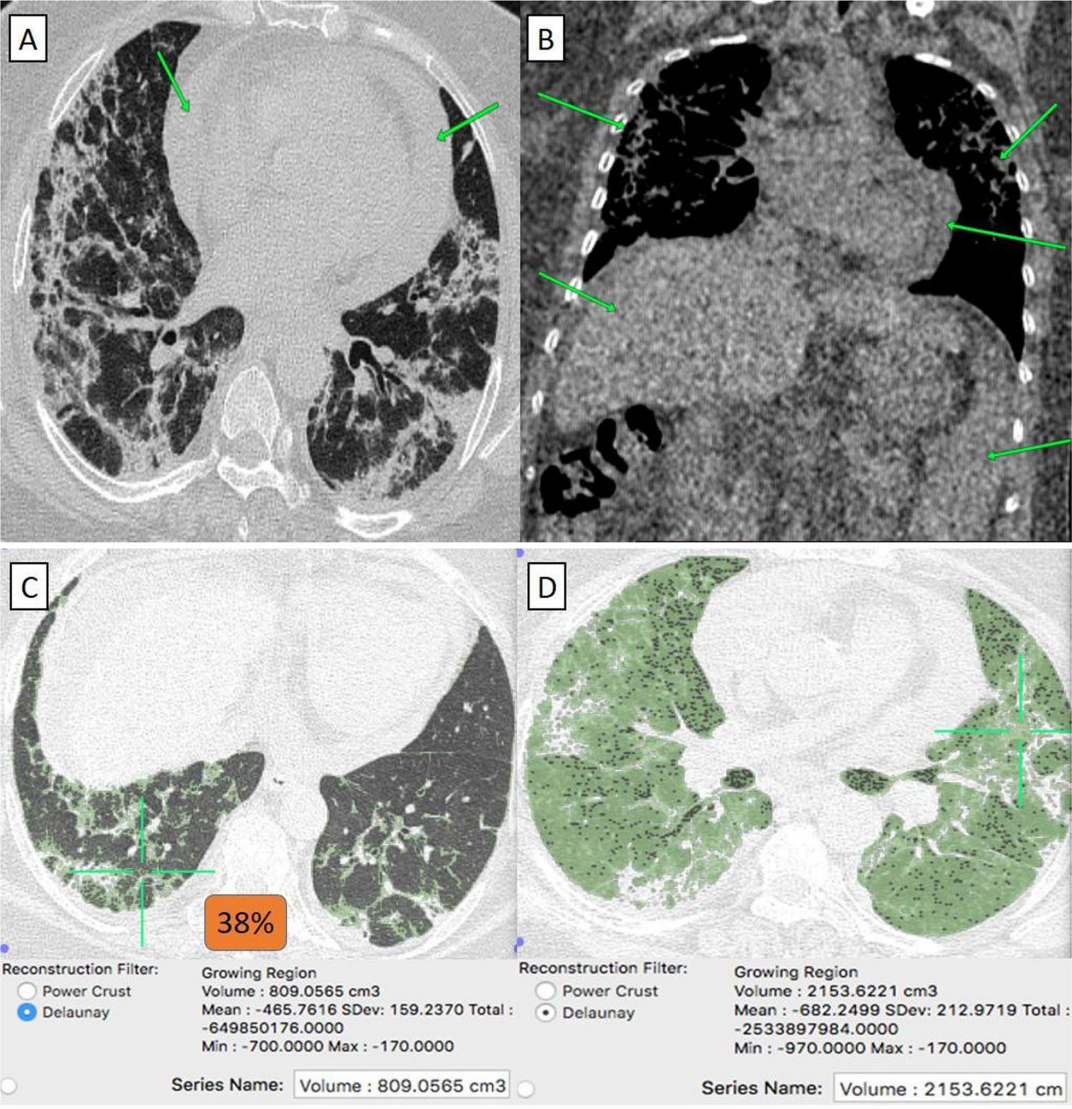
A 57-year-old female cardiac patient with history of HCV-related liver cirrhosis and had received “Oxford-AstraZeneca” vaccine then got COVID-19 infection. **A** Axial chest CT cut (lung window) showed bilateral mainly peripheral located heterogeneous ground-glass pneumonic patches with coarse septal thickening “patchy crazy-paving pattern” and large amount of pericardial collection (green arrows). **B** Coronal chest and upper abdomen CT cuts (mediastinal window) showed peripheral pneumonic patches, pericardial collection, liver cirrhosis and splenomegaly (arrows) **C** CT-Volumetry of the pathological lung parts averages 809 cc. [D] CT-Volumetry of total lung volume averages 2153 cc. So, 38% of the lung parenchyma was diseased (Score 2)

They were 12/55 (22%) patients. Their age ranged from 41 to 70 years with a male-to-female ratio of 1:3. The classification of comorbidities was described in (Table 1). Clinically, 11/12 (91%) patients were mild. By CT-volumetry, 7 (58%) patients scored (0) and 4 (33%) patients scored (1). Meanwhile, 1/12 (9%) patients was clinically severe, showed a "crazy paving pattern" in HRCT, were admitted to the hospital, and scored (2) (Fig. 8). (Tables 1, 2).

#### "Pfizer-BioNTech" received patients

**Fig. 9:**
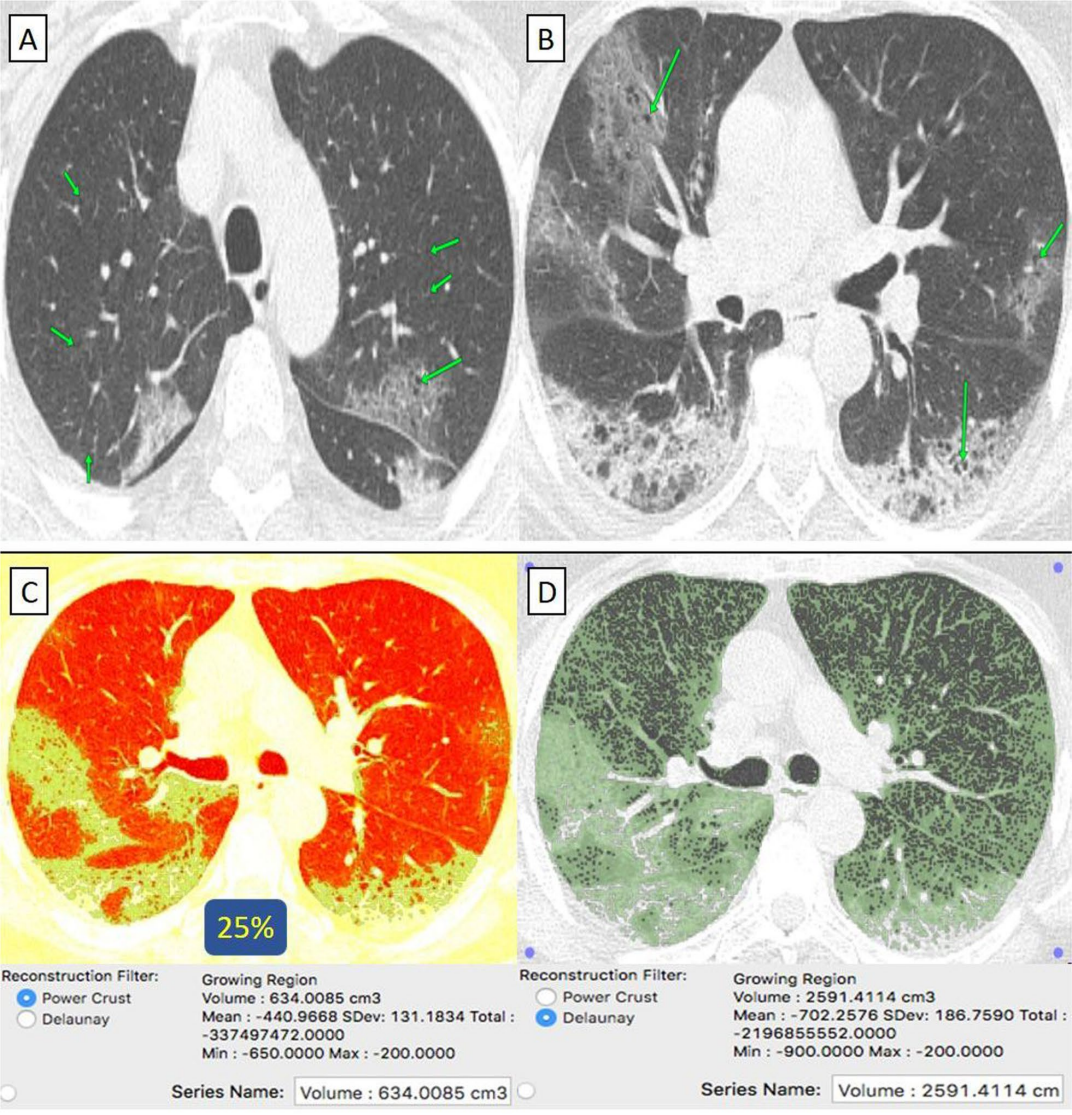
A 75-year-old male patient with history of lung emphysema and had received “Phizer-Biontech” vaccine then got COVID-19 infection. **A**, **B** Axial chest CT cuts (lung window) showed bilateral centrilobular emphysema as well as peripheral located and to lesser extent bronchocentric ground-glass pneumonic patches with cystic changes. **C** CT-Volumetry of the pathological lung parts averages 634 cc. **D** CT-Volumetry of total lung volume averages 2591 cc. So, 25% of the lung parenchyma was diseased (Score 1)

They were 6/38 (16%) patients. Their age ranged from 43 to 69 years with a male-to-female ratio of 1:2. (Table 1) All patients were clinically mild. By CT-volumetry, four (67%) patients scored (0) and two (33%) patients scored (1) (Fig. 9). (Tables 1, 2).

### Immunocompromised patients with a history of malignancy

The "Phizer-Biontech" vaccine, the "Oxford-AstraZeneca" vaccine, and to a lesser extent the "Sinopharm" vaccine had decreased the clinical severity in the immunocompromised infected patients (averaged 17%, 20%, and 50% respectively), compared to 79% severity in the non-immunized patients.

The CT severity scores also declined in the "Phizer-Biontech" and the "Oxford-AstraZeneca" vaccines' received patients (score 2 at both), and to a lesser extent in the "Sinopharm" received patients (score 2–3) in comparison to the non-immunized patients (reached score 4).

This is detailed as follows:

#### Non-immunized patients (control group)

They were 14/156 (90%) patients. Their age ranged from 21 to 67 years with a male-to-female ratio of 2:3. (Table 1) Clinically, 3/14 (21%) patients of them were mild and scored (1) by CT-volumetry. Meanwhile, 11/14 (79%) patients were clinically severe or critical, showed a "crazy paving pattern" in HRCT, and were admitted to the hospital. Five (36%) patients scored (2), four (29%) patients scored (3) & two (15%) patients scored (4). (Tables 1, 2).

#### "Sinophram" received patients

**Fig. 10:**
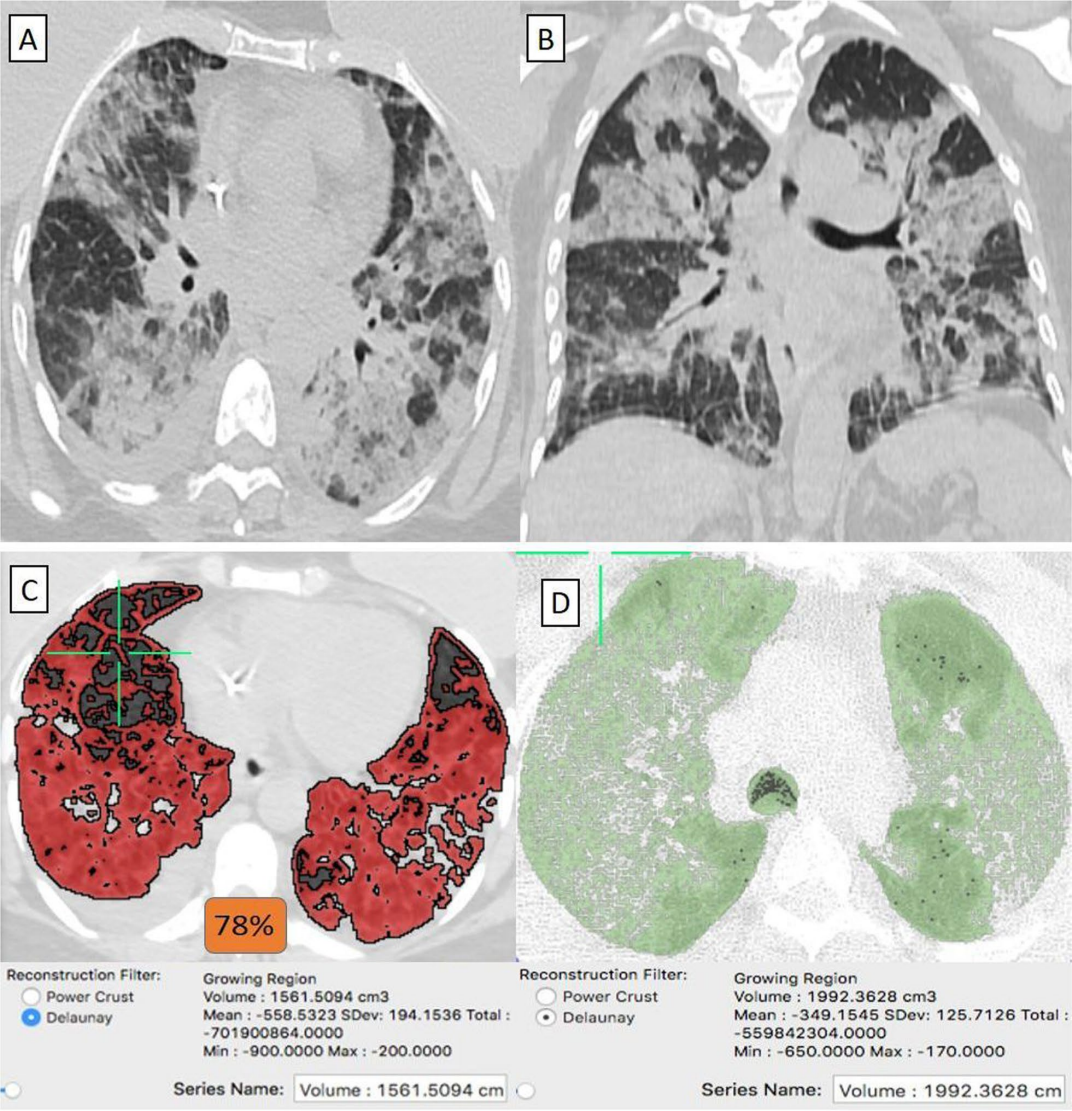
A 53-year-old female patient with history of operated bilateral breast cancer and had received “Sinopharm” vaccine then got COVID-19 infection. **A** Axial, and **B** Coronal chest CT cuts (lung window) showed bilateral wide spread large heterogeneous ground-glass patches with consolidative changes and minimal right pleural collection. Breast implants are also noticed in axial cuts. **C** CT-Volumetry of the pathological lung parts averages 1561 cc. **D** CT-Volumetry of total lung volume averages 1992 cc. So, 78% of the lung parenchyma was diseased (Score 4)

They were 10/92 (11%) patients. Their age ranged from 34 to 79 years with a male-to-female ratio of 1:4. (Table 1) Clinically, 5/10 (50%) patients were mild. By CT-volumetry, 1 (10%) patient scored (0) and 4 (40%) patients scored (1). Meanwhile, 5/10 (50%) patients were clinically severe or critical, showed a "crazy paving pattern" in HRCT, and were admitted to the hospital. Two (20%) patients scored (2), two (20%) patients scored (3), and one (10%) patient scored (4) (Fig. 10). (Tables 1, 2).

#### "Oxford-AstraZeneca" received patients

They were 5/55 (9%) patients. Their age ranged from 41 to 76 years with a male-to-female ratio of 1:4. (Table 1) Clinically, 4/5 (80%) patients were mild. By CT-volumetry, 3 (60%) patients scored (0) and one (20%) patient scored (1). Meanwhile, 1/5 (20%) patients was clinically severe, showed a "crazy paving pattern" in HRCT, were admitted to the hospital, and scored (2). (Tables 1, 2).

#### "Pfizer-BioNTech" received patients

They were 6/38 (16%) patients. Their age ranged from 39 to 73 years with a male-to-female ratio of 2:3. (Table 1) Clinically, 5/6 (83%) patients were mild. By CT-volumetry, 4 (67%) patients scored (0) and one (17%) patient scored (1). Meanwhile, 1/6 (17%) patients was clinically severe, showed a "crazy paving pattern" in HRCT, were admitted to the hospital, and scored (2). (Tables 1, 2).

### Comparative efficacy [analysis of variance (ANOVA) with Tukey HSD/Tukey Kramer]: (Table 3)

**Table 3:** Comparative analysis of variance (ANOVA) results

	Sinopharm versus non-vaccinated	Ox-AstraZeneca versus non-vaccinated	Pfizer-BioNTech versus non-vaccinated	Ox-AstraZeneca versus Sinopharm	Pfizer-BioNTech versus Sinopharm	Pfizer-BioNTech versus Ox-AstraZeneca
*Patients with good general health and absent co-morbidity*						
F-ratio	4.0282	10.8049	8.4404	2.1473	1.8586	0.003984
*p*-value	**0.04632** **(Significant)**	**0.001268** **(Significant)**	**0.004294** **(Significant)**	0.146 (Non-significant)	0.1763 (Non-significant)	0.9499 (Non-significant)
Effect size	0.15 (Small)	0.27 (Medium)	0.25 (Medium)	0.15 (Small)	0.15 (Small)	0.008 (Small)
η2 (variance from average)	0.023 (2.3%)	0.069 (6.9%)	0.059 (5.9%)	0.021 (2.1%)	0.021 (2.1%)	0.000064 (0.006%)
Tukey HSD / Tukey Kramer	**Significant** (× 1– × 2	**Significant** (× 1– × 2)	**Significant** (× 1– × 2)	Non-significant	Non-significant	Non-significant
*Patients with chronic co-morbidity*						
F-ratio	0.1784	4.7521	4.1682	5.4522	6.5531	0.2807
*p*-value	0.6746 (Non-significant)	**0.03492** **(Significant)**	**0.04857** **(Significant)**	**0.02667** **(Significant)**	**0.01751** **(Significant)**	0.6035 (Non-significant)
Effect size	0.06 (Small)	0.34 (Large)	0.34 (Large)	0.43 (Large)	0.53 (Large)	0.13 (Small)
η2 (variance from average)	0.0036 (0.04%)	0.1 (10.2%)	0.1 (10.4%)	0.16 (15.8%)	0.22 (22.2%)	0.017 (1.7%)
Tukey HSD / Tukey Kramer	Non-significant	**Significant** (× 1– × 2)	**Significant** (× 1– × 2)	**Significant** (× 1– × 2)	**Significant** (× 1– × 2)	Non-significant
*Immunocompromised patients*						
F-ratio	1.4856	11.7811	15.6	3.7143	5.1886	0.03664
*p*-value	0.2358 (Non-significant)	**0.003177** **(Significant)**	**0.0009394** **(Significant)**	**0.07609** **(Significant)**	**0.03895** **(Significant)**	0.8525 (Non-significant)
Effect size	0.26 (Medium)	0.83 (Large)	0.93 (Large)	0.53 (Large)	0.61 (Large)	0.064 (Small)
η2 (variance from average)	0.063 (6.3%)	0.41 (40.9%)	0.46 (46.4%)	0.22 (22.2%)	0.27 (27%)	0.0041 (0.4%)
Tukey HSD / Tukey Kramer	Non-significant	**Significant** (× 1– × 2)	**Significant** (× 1– × 2)	**Significant** (× 1– × 2)	**Significant** (× 1– × 2)	Non-significant
*Globally*						
F-ratio	2.6941	18.5913	15.6817	8.43294	7.97616	0.1092
*p*-value	0.102 (Non-significant)	**0.00002495** **(Significant)**	**0.0001055** **(Significant)**	**0.004262** **(Significant)**	**0.005499** **(Significant)**	0.741812 (Non-significant)
Tukey HSD / Tukey Kramer	Non-significant	**Significant** (× 1– × 2)	**Significant** (× 1– × 2)	**Significant** (× 1– × 2)	**Significant** (× 1– × 2)	Non-significant

*p*-value (< 0.05) was considered statistically significant

#### In patients with good general health and absent co-morbidity

In addition to the previously mentioned efficacy results based on the prevalence and ratio of the clinical and the CT-severity scores, the ANOVA testing with Tukey HSD / Tukey Kramer revealed significant variance regarding the use of the "Sinopharm", the "Oxford-AstraZeneca", and the "Phizer-Biontech" vaccines in comparison to the non-immunized patients (*f*-ratio averaged 4.0282, 10.8049, and 8.4404 respectively, also *p*-value averaged 0.04632, 0.001268, and 0.004294, all < 0.05). (Table 3).

Meanwhile, no significant variance was proved between the use of either type of the above-mentioned vaccines in comparison to the rest. (Table 3).

#### In patients with co-morbidities

In addition to the previously mentioned efficacy results based on the prevalence and ratio of the clinical and the CT-severity scores, the ANOVA testing with Tukey HSD / Tukey Kramer revealed significant variance regarding the use of the "Oxford-AstraZeneca", and the "Phizer-Biontech" vaccines in comparison to the non-immunized patients (*f*-ratio averaged 4.7521, and 4.1682 respectively, also *p*-value averaged 0.03492, and 0.04857, both < 0.05). (Table 3).

On the other hand, and despite the percentages of the decline of the clinical severity, the results of ANOVA testing (*f*-ratio averaged 0.1784, and *p*-value averaged 0.6746 > 0.05) did not show significant variance regarding the use of the "Sinopharm" vaccine. (Table 3).

Consequently, a significant variance was proved between the use of the "Oxford-AstraZeneca" and the "Phizer-Biontech" vaccines in comparison to the "Sinopharm" vaccine. Meanwhile, no significant variance was proved between the use of the "Oxford-AstraZeneca" against the "Phizer-Biontech" vaccines themselves. (Table 3).

#### In immunocompromised patients

In addition to the previously mentioned efficacy results based on the prevalence and ratio of the clinical and the CT-severity scores, the ANOVA testing with Tukey HSD / Tukey Kramer revealed significant variance regarding the use of the "Oxford-AstraZeneca", and the "Phizer-Biontech" vaccines in comparison to the non-immunized patients (*f*-ratio averaged 11.7811, and 15.6 respectively, also *p*-value averaged 0.003177, and 0.0009394, both < 0.05). (Table 3).

On the other hand, and despite the percentages of the decline of the clinical severity, the results of ANOVA testing (*f*-ratio averaged 1.4856, and *p*-value averaged 0.2358 > 0.05) did not show significant variance regarding the use of the "Sinopharm" vaccine. (Table 3).

Again, a significant variance was proved between the use of the "Oxford-AstraZeneca" and the "Phizer-Biontech" vaccines in comparison to the "Sinopharm" vaccine. Meanwhile, no significant variance was proved between the use of the "Oxford-AstraZeneca" against the "Phizer-Biontech" vaccines themselves. (Table 3).

#### Globally in all patients

The ANOVA testing with Tukey HSD / Tukey Kramer revealed significant variance regarding the use of the "Oxford-AstraZeneca", and the "Phizer-Biontech" vaccines in comparison to the non-immunized patients (*f*-ratio averaged 18.5913, and 15.6817 respectively, also *p*-value averaged 0.00002495, and 0.0001055, both < 0.05). (Table 3).

On the other hand, and despite the percentages of the decline of the clinical severity, the results of ANOVA testing (*f*-ratio averaged 2.6941, and *p*-value averaged 0.102 > 0.05) did not show significant variance regarding the use of the "Sinopharm" vaccine. (Table 3).

Consequently, a significant variance was proved between the use of the "Oxford-AstraZeneca" and the "Phizer-Biontech" vaccines in comparison to the "Sinopharm" vaccine. Meanwhile, no significant variance was proved between the use of the "Oxford-AstraZeneca" against the "Phizer-Biontech" vaccines themselves. (Table 3).

## Discussion

Several factors are currently responsible for the reported incidences of COVID-19 breakthrough infections. They included the percentage of vaccinated people, the type of available vaccine, the duration elapsed since vaccination, and the viral variants [11–14].

Several clinical-laboratory pieces of research compared the efficacy of different vaccines in different countries all over the world but very few researches were performed on the radiological role. This may be attributed to the fact that the need for chest CT examinations had much regressed after the availability of vaccinations, particularly among the asymptomatic and mildly symptomatic patients. Consequently, a longer duration is acquired to obtain a reasonable cohort that includes patients of different age groups, different medical histories, and of course different clinical courses and outcomes.

In this study, all symptomatic patients were eligible for chest CT examinations based on their work regulations, particularly health workers and co-workers. Also, the asymptomatic patients were eligible for chest CT examinations before hospitalization for other causes than COVID-19 infection. Eventually, this increased the variations within the selected cohort and decreased its bias. In the current experience, the authors compared three widely distributed different kinds of COVID-19 vaccinations applied in their country.

According to Olliaro P et al. [15], the efficacy of the vaccination was measured by comparing the attack rates without and with vaccinations, referred to as “relative risk reduction". Many other pieces of research correlated the efficacy of the vaccination to the clinical outcome of the disease, particularly regarding the percentage of hospitalization, the percentage of intense care unit admission, and the total hospital stay. They correlated the history of vaccination with the risk of reduction manifested by the less likely disease progression, need for hospitalization, or mechanical ventilation.

Tenforde MW et al. [16] clinical study included 314 vaccinated and 1669 control COVID-19 patients compared to 185 and 156 patients in this study respectively. The clinical severity in their study accounted for 15.8–21.9% and 54.8–61.8% in the vaccinated and non-vaccinated patients respectively compared to 7 and 12.1% in this study. The higher percentage of clinical severity in their study is attributed to the predominance of alpha and delta variants of the virus, meanwhile, this study was performed during the predominance of delta and omicron variants. The hospitalization in their study was four times increased in the immunocompromised patients compared to the ten times increase in this study.

This study matched Karimi M et al. [17] clinical study on vaccinated COVID-19 patients with hemoglobinopathies which reported the disease severity in 7% of their patients compared to 8% of patients with variable comorbidities in this study.

This study also agreed with Edan MH et al. [18] clinical study regarding the stronger immunity given by the “Phizer-BionTech” vaccine compared to the “Sinopharm” vaccine.

The results of the Al-Khazrajy DF et al. [19] clinical study almost matched the results of this study regarding the presence of significant variance between immunity of the “Phizer-BionTech” and the “AstraZeneca” vaccinated patients versus the non-vaccinated control patients. Their study generally found no significant variance between the “Sinopharm” vaccinated patients compared to the non-vaccinated control patients. The current study restricted this result to the patients with morbidities and immunocompromised patients, meanwhile, a significant variance was detected in the vaccinated patients without comorbidities.

The results of the current study mismatched that of Ghiasi N et al. [20] clinical study which reported 100% protection from clinical severity in “Sinopharm” vaccinated patients compared to a general 90% in this study. The current study furtherly classified this protection to be 97% in patients without comorbidities, 90% in patients with comorbidities, and 50% in immunocompromised patients.

Very few studies utilized the quantitative and qualitative role of CT. They used the original semi-quantitative method of pulmonary lobar sections’ division.

Lee JE et al. [21] reported positive chest CT examinations with pneumonia in 78% and 41% of non-vaccinated and vaccinated patients respectively, compared to 81% and 55% in this study. Furthermore, this study particularly agrees with Modi SD et al. [22] regarding the low CT severity scores in the vaccinated patients with comorbidities compared to the non-vaccinated patients. It also matches similar general results of Madhu P et al. [2] study.

This study added to the literature the novel use of quantitative volumetric CT results instead of the previous semi-quantitative methods. The other advantage of this study was the detailed classification of patients according to the presence or absence of comorbidities or immunocompromised status. It benefited the utilization of the volumetric CT-scoring lowering rates side by side with the ANOVA results for more accurate results. Bias was reduced as possible by widening the selection criteria to include the asymptomatic and mildly symptomatic patients. Additionally, it correlated the results to the clinical severity and morphologic CT signs of severity such as the “crazy-paving pattern”.

The main limitation of this study was the moderate number of included patients, also the data about the virus variants were deficient. Therefore, further larger group research with detailed knowledge is recommended.

This study did not target the effectiveness or safety of the vaccinations which should be evaluated by other dedicated professional researchers.

## Conclusions

This study added to the literature the novel use of quantitative volumetric CT results instead of the previous semi-quantitative methods side by side with ANOVA.

In addition to the decline of clinical severity rates & CT severity scores, a significant variance was proved regarding the use of the "Sinopharm", "Oxford-AstraZeneca", and "Phizer-Biontech" vaccines in patients without comorbidities.

Significant variance was also proved regarding the use of the "Oxford-AstraZeneca" and "Phizer-Biontech" vaccines in patients with comorbidities and immunocompromised patients.

Despite that, no significant variance could be proved regarding the use of the "Sinopharm" vaccine in these patients, it decreases the percentage of clinical severity and CT severity scores.

## Data Availability

The datasets used and/or analyzed during the current study are available from the corresponding author on reasonable request.

## References

[1] Sonali DM, Dharita SS, Krati SM (2021). Comparative study of CT severity index and outcome in hospitalized vaccinated and non-vaccinated patients of Covid 19 pneumonia. J Radiol Clin Imaging.

[2] Madhu P, Santhosh D, Kiran M (2021). Comparison study of lung involvement in vaccinated and unvaccinated covid patients. Int J Health Clin Res.

[3] Kanne JP, Bai H, Bernheim A (2021). COVID-19 imaging: what we know now and what remains unknown. Radiology.

[4] Hodgson SH, Mansatta K, Mallett G, Harris V, Emary KR, Pollard AJ (2021). What defines an efficacious COVID-19 vaccine? A review of the challenges assessing the clinical efficacy of vaccines against SARS-CoV-2. Lancet Infect Dis.

[5] Brogna B, Bignardi E, Brogna C (2021). Covid-19 pneumonia in vaccinated population: a six clinical and radiological case series. Medicina (Lithuania).

[6] Dooling K, McClung N, Chamberland M, Oliver S (2020). The advisory committee on immunization practices’ interim recommendation for allocating initial supplies of COVID-19 vaccine—United States, 2020. MMWR Morb Mortal Wkly Rep.

[7] Adam L, Rosenbaum P, Bonduelle O, Combadière B (2021). Strategies for immunomonitoring after vaccination and during infection. Vaccines.

[8] Voysey M, Clemens SA, Madhi SA (2021). Safety and efficacy of the ChAdOx1 nCoV-19 vaccine (AZD1222) against SARS-CoV-2: an interim analysis of four randomised controlled trials in Brazil, South Africa, and the UK. Lancet.

[9] Katal S, Pouraryan A, Gholamrezanezhad A (2021). COVID-19 vaccine is here: practical considerations for clinical imaging applications. Clin Imaging.

[10] Chen LYC, Hoiland RL, Stukas S (2020). Confronting the controversy: interleukin-6 and the COVID-19 cytokine storm syndrome. Eur Respir J.

[11] Bergwerk M, Gonen T, Lustig Y (2021). Covid-19 breakthrough infections in vaccinated health care workers. N Engl J Med.

[12] Brinkley-Rubinstein L, Peterson M, Martin R, Chan P, Berk J (2021). Breakthrough SARS-CoV-2 infections in prison after vaccination. N Engl J Med.

[13] Hacisuleyman E, Hale C, Saito Y (2021). Vaccine breakthrough infections with SARS-CoV-2 variants. N Engl J Med.

[14] Thompson MG, Grant L, Meece JHEROES-RECOVER Network. Prevention of Covid-19 with the BNT162b2 and mRNA-1273 Vaccines. Reply. N Engl J Med. 2021;385(19):1819–1821. 10.1056/NEJMc2113575 34614323

[15] Olliaro P, Torreele E, Vaillant M (2021). COVID-19 vaccine efficacy and effectiveness—the elephant (not) in the room. Lancet Microbe.

[16] Tenforde MW, Self WH, Adams K (2021). Association between mRNA vaccination and COVID-19 hospitalization and disease severity. JAMA.

[17] Karimi M, Zarei T, Haghpanah S (2022). Efficacy and safety of sinopharm vaccine for SARS-CoV-2 and breakthrough infections in iranian patients with hemoglobinopathies: a preliminary report. Mediterr J Hematol Infect Dis.

[18] Edan MH, Khalaf YH, Geeran AM (2022). Pfizer-BioNTech and Sinopharm: a comparative study on biochemical and immunological responses in healthy individuals post-vaccination against COVID-19. J Emerg Med Trauma Acute Care.

[19] Al-Khazrajy DF, Raddam QN (2022). Evaluation of the efficacy of COVID-19 vaccines (Pfizer, Astra Zeneca, Sinopharm) using iraqi local samples. NeuroQuantology.

[20] Ghiasi N, Valizadeh R, Arabsorkhi M (2021). Efficacy and side effects of Sputnik V, Sinopharm and AstraZeneca vaccines to stop COVID-19; a review and discussion. Immunopathol Persa.

[21] Lee JE, Hwang M, Kim YH (2022). Imaging and clinical features of COVID-19 breakthrough infections: a multicenter study. Radiology.

[22] Modi SD, Shah DS, Mundhra KS (2021). Comparative study of CT severity index and outcome in hospitalised vaccinated and non vaccinated patients of Covid 19 pneumonia. J Radiol Clin Imaging.

